# Physicians’ experiences of video consultation with patients at a public virtual primary care clinic: a qualitative interview study

**DOI:** 10.1080/02813432.2021.1882082

**Published:** 2021-03-02

**Authors:** Cajsa Björndell, Åsa Premberg

**Affiliations:** a Närhälsan Brämaregården Health Centre, Gothenburg, Sweden; b Primary Health Care/School of Public Health and Community Medicine, Institute of Medicine, Sahlgrenska Academy, University of Gothenburg, Gothenburg, Sweden; c Research and Development Primary Health Care, Research and Development Centre Gothenburg and Södra Bohuslän, Gothenburg, Sweden; d Institute of Health and Care Sciences, Sahlgrenska Academy, University of Gothenburg, Gothenburg, Sweden

**Keywords:** Primary health care, general practise, video consultation, virtual care, telemedicine, consultation process, qualitative research

## Abstract

**Objective:**

To describe physicians’ experiences of video consultation with new patients visiting a publicly owned virtual primary care clinic.

**Design:**

In this qualitative study, data were collected from semi-structured individual interviews and analysed by systematic text condensation.

**Setting:**

A publicly owned virtual primary care clinic in Region Västra Götaland, Sweden.

**Subjects:**

Ten primary care physicians working at the clinic.

**Results:**

Connecting with a patient over video could be either straightforward or deficient, depending on communication and the patient’s condition. Clinical experience, communication skills, and involving patients throughout the consultation and examination were crucial for assessments over video where patients were guided to perform self-examination. The flexibility of work and the regulated assignment online were positive for the physicians’ work situation and wellbeing. Providing video consultation within the same organisation as the patient’s regular health centre was considered to facilitate patient care and safety. Video consultation was considered suitable for some diagnoses and for some patients not able to reach a primary healthcare centre, though doubts were expressed about the healthcare and social benefits of this virtual care service.

**Conclusion:**

For the physicians, video consultation induced changes in the basis for assessment of primary care patients. The limitations on informational exchange demanded an extended form of patient involvement founded upon consultation skills, clinical experience and new skills for virtual examination. Combining virtual care with traditional general practice has the potential to reduce the workload for the individual physician and ensure medical competence in virtual primary care. Video consultation experienced suitable in some situations, but easy access to it expressed problematic in terms of medical prioritisation in healthcare.

**KEY POINTS**
Video consultation is suitable for primary care visits for some patients, but physicians’ experiences of this are rarely studied.
•Clinical experience and consultation skills are important for video assessment of primary care patients which involves physician-guided patient self-examination.
•Video consultation facilitates care in some situations and could benefit from the provider being connected to patient’s regular health centre.
•Virtual care offers a flexible way of working but challenges healthcare prioritisation from the primary care physician’s perspective.

## Introduction

Digitalisation has produced radical developments in healthcare, offering several alternatives to in-person meetings between patient and physician. In Sweden, consultations with primary care physicians by digital platforms has increased around 20% per month after its introduction in mid-2016 [1]. Video consultation,which in this context means a virtual care service with synchronous video-mediated patient-physician consultation is increasingly used in primary care. It is a vital part of telemedicine or telehealth, which are synonymous and broader terms referring to all kind of healthcare services over distance, and consequently increasing the accessibility of healthcare [2–5]. The coronavirus pandemic has made telemedicine even more central in healthcare [6]; in particular, virtual care where video consultation has been scaled up in order to reduce the transmission risk [3,7]. Nevertheless, it is still not fully clear how well video consultation works in general and for physicians in particular [4,5].

Previous research on video consultation in primary care has mainly focused on the patient perspective [8–12], follow-up care [9,11,13,14], and on virtual care services provided by private companies or in countries where healthcare is mainly financed through private health insurance [8–10,12,13,15,16]. Video consultation is convenient and time saving for patients [8,9,12,14], who appreciate its high availability [12]. However, the only qualitative study found, including physicians’ experiences of video consultation, was focused on follow-ups and the importance of an existing doctor-patient relationship when conducting video consultation [14]. Furthermore, there are only a few systematic reviews in this area, and their contribution to knowledge of video consultation with patients in primary care is limited [17,18].

In comparison with the existing literature, the context and object of this study are rare: a publicly owned virtual primary care clinic where video consultation can be used for the initial contact with new patients. We chose to take advantage of the physicians’ knowledge and experience, and so the aim of this study was to describe physicians’ experiences of video consultation with new patients visiting a publicly owned virtual primary care clinic.

## Material and methods

A qualitative method was chosen in order to allow exploratory work, since the knowledge of the phenomenon is limited. Semi-structured interviews were used to gain a deeper understanding of the physicians’ individual experiences, and the material was analysed by systematic text condensation according to Malterud, inspired by Giorgi [19,20]. This method is mainly based on phenomenology, which is appropriate for a descriptive transverse analysis aimed at developing knowledge of the participants’ experiences.

### Setting and participants

This study was conducted in Region Västra Götaland in south-west Sweden. The majority of the population of the region live in urban areas, and aside from those living on the archipelago, over 80% have less than 5 min’ drive on average to the nearest health centre [21]. In Sweden consultations with primary care physicians by digital platforms is most commonly used in urban areas, and mainly provided by for-profit private firms [1]. Närhälsan Online is the virtual clinic of the public primary care provider Närhälsan, run by the region. With extended opening hours, Närhälsan Online provides video consultation as a separate virtual care service to all of Närhälsan’s 104 health centres. Patients use an application to consult a general practitioner or a resident in general practice through a video call without involvement of a third party. At the time of data collection, there was no prior triage at the virtual primary care clinic. The patients booked the appointments themselves, although a majority had been recommended to book a video consultation by telephone triage nurses at their health centres. Video consultations were scheduled at 20-minute intervals, and patients were free to upload images and specify the reason for the consultation when booking [22].

The first author recruited participants by email, or by phone after they had received information about the study in an email. Of the 26 physicians who met the inclusion criterion of a minimum 25 h’ working time at Närhälsan Online, one did not meet the other criterion of 2.5 years’ experience in general practice and two were excluded because they were personally known by the project leader. In order to achieve a variation in the material, a strategic selection was made on the basis of gender, profession, experience, workplace, medical degree and birth country. Ten physicians were interviewed (Table 1).

**Table 1.** Demographic data of participants (*n* = 10).

Age (years)	
Median	41.5
Range	29–54
Gender (*n*)	
Female	5
Male	5
Profession (*n*)	
General practitioner	5
Resident in general practice	5
Country of birth (*n*)	
Sweden	4
Other European country	3
Non-European country	3
Place of medical degree (*n*)	
Sweden	5
Other country	5
Ordinary workplace, municipality group* (*n*)	
Large city	1
Commuting municipality near large city	5
Larger city	3
Commuting municipality near larger city	1
Experience in primary care (years)	
Median	5.5
Range	3–20
Experience with video consultation at Närhälsan Online (clinical hours)	
Median	150
Range	30–451

*Municipality group division according to the Swedish Association of Local Authorities and Regions.

### Data collection

The first author conducted the interviews between September 2018 and January 2019. Each interview lasted between 49 and 72 min (median 60 min) and was audio recorded. Participants were interviewed during their working hours, at their workplace or at home. The first interview was a pre-test and was included in the study because no substantial changes were made in the interview technique. A semi-structured interview guide was used; the main question was ‘Please tell me what it’s been like to work with video consultation at the virtual primary care clinic’, and open-ended and clarifying questions were used to invite the participants to deepen their experiences, such as ‘Please tell me more’ and ‘How do you mean?’. Summaries of what the participant had been talking about were also used to move the conversation forward. After nine interviews no further information was revealed, and the data collection was concluded after ten interviews. The interviews were transcribed literally; the first by the first author and the rest by medical secretaries. In order to correct any misunderstandings, the first author scrutinised all the transcriptions, and one was also carefully read by the participant herself at her request. This resulted in minor corrections.

### Data analysis

The authors performed the analysis in collaboration through four steps. First, the transcripts were read through three times to gain an overall impression. Second, units of meaning corresponding to the aim of the study were manually identified and sorted, followed by a systematic decontextualisation where the texts were lifted out of context and matched with related text elements. Third, the resulting code groups were condensed into concrete content. Fourth, a recontextualisation was performed in which the content of each code group was described and summarised and an analytical text with categories and subcategories was written [19].

## Results

Connecting with a patient over video could be either straightforward or deficient, depending on communication and the patient’s condition. Clinical experience, communication skills, and involving patients throughout the consultation and examination were crucial for assessments over video where patients were guided to perform self-examination. The flexibility of work and the regulated assignment online were positive for the physicians’ work situation and wellbeing. Providing video consultation within the same organisation as the patient’s regular health centre was considered to facilitate patient care and safety. Video consultation was considered suitable for some diagnoses and for some patients not able to reach a primary healthcare centre, though doubts were expressed about the healthcare and social benefits of the virtual care service. Below, we elaborate these findings with selected quotations. The participants (P) are numbered in random order.

### Connecting through the screen: face to face at the patient’s place with just one click

Patients were perceived to be relaxed in their own environment, but were sometimes unaccustomed to the contact with the physicians over video; this needed to be handled, but could also act as a conversation opener. The insight into patients’ daily lives brought a feeling of getting closer to the patients. The patients were described as being happy, pleasant, and friendly, as if this was something unusual. This favoured connection with the patient as well as the physicians’ well-being.

‘Oh, yes… I’m lying here in bed with a fever’… it’s a bit special, you get into their private life in a way that you don’t do here [at the health centre]… sometimes things can get a bit giggly, and sometimes it can get a bit stiff … (P 2)

The main difference between patients I meet at the health centre and online is that the patients you meet online are very satisfied…they are incredibly happy to be able to talk to a doctor… in a comfortable environment… (P 1)

Reported prerequisites for connecting with the patient through video were good consultation technique, absence of technical problems, absence of people in the background, and a stable mental status of the patient. It was difficult if a patient was strongly mentally affected during the consultation and disappeared from the screen. Under optimal conditions, the contact was similar to a physical meeting, and was described with expressions usually related to physical meetings such as ‘eye contact’ and ‘face to face’. Starting and ending the consultation with a click on the screen was perceived as being efficient and as facilitating clinical focus. It felt easy to end the conversation, even when no agreement with the patient was achieved.

… some patients with very strong mental illness, especially those who are very depressed… I suddenly don’t see them, ‘Are you still here?’ They’re sitting, shaking, with the phone in their hands … (P 8)

It’s easier to end the consultation online, the patient doesn’t have to get dressed… it goes very quickly … you can be more efficient and there’s more room for specific issues as well. You can be more defined in the contact. (P 9)

### See, hear, and trust the patient: strategies for assessment over video

Listening to the patient’s thoughts, concerns, and requests was seen as both time-efficient and crucial for prioritisation, decision making, and outcome. Some of the physicians asked the patients various questions in an attempt to be time-efficient or to achieve a reliable assessment despite the absence of physical examination. One of them spoke about having to assess a skin lesion on the basis of anamnesis, because the image of the lesion was blurry.

…you can ask the patient, ‘How do you want me to help you with this?’ and you might get ‘I think this is on the way out, but now I just need sick leave for two days because I can’t work yet’ … then you can feel assured that there’s no need for any more physical assessment. (P 7)

The physicians sometimes guided the patients through a self-examination during the video consultation; for example, to perform a self-palpation. Their assessment could be based on both their own findings and the patient’s findings; this required them to trust the patient, which could feel doubtful. It could also feel difficult to trust the patient when it came to sick notes. A common strategy was to avoid doing anything online that did not feel safe, but instead refer the patient to a physical care unit. Clinical experience from traditional primary care was highlighted as particularly important to enable an accurate assessment despite incomplete information. Some considered it judicious to refer the patient for a physical examination if the case did not cohere with similar previous cases, to avoid missing unusual illnesses.

‘How does the skin feel when you touch it, soft or hard?’ or ‘Can you move the lump?’ … So you really have to guide the patient through the physical examination… (P 4)

… you don’t want to think someone’s staying home even though they’re able to work, but you still have this gnawing thought that this might be the case… Probably I’d have found it difficult to estimate how long it would last if I’d met them physically, but it’s even more difficult with video consultations… fewer objective findings. (P 8)

… you’ve seen many of these ailments in real life at the clinics… you learn to use fewer senses virtually than you do out at the health centre. (P 10)

### A new work situation: many roles but gained flexibility and reduced workload

Duties beside the traditional physician’s role, such as triage, technical assistance, and guidance, characterised the work. One of the physicians expressed that it was fun to work as a nurse when booking appointments at health centres for patients needing physical examination. However, not being able to help the patients through video consultation could bring feelings of wasting time, frustration, and not making any difference. Guiding patients in how to handle the camcorder and get internet access were described as regular but creative parts of the visit. The outcome of the visit could depend on the patient’s technical skills, and some consultations had to be ended because of the patient’s technical problems. It was also surprising how well the technology worked out.

As a doctor online, you combine the role of triage nurse but also treating doctor… you can also be your own secretary… and you’re also your own IT support if something goes wrong… Many roles in one. (P 10)

… the video call freezes and they disappear and you…‘Turn off your WIFI then, connect on 4G…’ Or they can’t connect the call, then you say they can restart the app and they don’t know how to do it, and you say, ‘Restart the whole phone instead’… then seven minutes of the visit has already passed. (P 7)

The work was experienced as being easier than ordinary work at the health centre, since the cases were simple and there was a strict framework that regulated the assignment online. One of them noted that patients raised fewer problems per visit compared to patients at the health centre, which they thought was due to the higher availability of appointments at the virtual primary care clinic. Some physicians found it a relief not to have to prescribe addictive drugs, perform follow-ups, or certify more than a week of sick leave. One of them found the absence of follow-up visits unstimulating, while another found the work stimulating due to the need for quick decision-making.

A shift at Närhälsan Online, especially if you work at home, it’s almost therapeutic… At the health centre, you’re like the last station for everything… some sort of dustbin for all issues… At Närhälsan Online you end up a little higher up… away from this, like a consultant… From a stress point of view, it’s a good thing. (P 9)

It’s a little limited in terms of follow-ups… a way of working that may not be as stimulating. I like to have long patient relationships, to follow people over time…. Then there are pretty simple things… where nursing advice and self-care would have been enough… it’s a bit under-stimulating… (P 5)

Working from home was appreciated by the physicians because it let them work in peace, feel less stressed, and enjoy being at home. The flexibility of working time and place made it possible to alternate work and leisure. Saving travel time, being present at home, and participating in family activities adjacent to the shift was considered beneficial in increasing quality of life. The reduced workload and quiet working environment made the physicians less stressed and increased their wellbeing; one physician who had been about to leave the profession said that this was crucial in her decision to remain in primary care, and that she was now able to work more than before.

I don’t get stressed driving in traffic, getting in here (at the health centre), turning on the computer and so on, then driving home… at home, when I’m done…I just log out and go down and watch TV or spend time with my family. (P 1)

### Doing some good online in times of barriers to primary care

The physicians had found that sometimes patients had booked the video consultation because they had not been able to get an appointment at their health centres. Because of the flexibility it offered in terms of distance and consultation hours, the virtual clinic was considered to facilitate medical consultation for patients who found it difficult to visit a health centre, such as families with children and patients with social phobia or panic disorder. Reduced risk of infection transmission was also mentioned as a benefit. The provision of video consultation within the same organisation as patient’s regular health centre, with a unified medical record, facilitated health information exchange and hence patient care and safety. Patients far from home, even abroad, were able to receive help through the virtual clinic.

… I remember a mother with a young child who had fallen at school and suffered a wound that had been taped, but the mother was still worried about the injury and it was in the evening and really cold outside … so it was great for her… to get a medical assessment in the evening from home. (P 3)

… a family was in Finland and had discovered that the child had erythema migrans and I had to write prescriptions that were faxed to the pharmacy in Finland. They got their penicillin… (P 2)

Skin rashes, eczema, and mild infections were perceived as generally easy to solve through video consultation. Some of the physicians considered it possible to assess skin tumours using the photos uploaded by the patients, while others considered this unsafe without palpation and dermatoscopy. Throat examinations could be difficult, but some doctors felt comfortable prescribing antibiotics against streptococcal tonsillitis based on the Centor criteria and the rapid laboratory test. Certification was a common task. One of the physicians highlighted the difficulty of excluding pneumonia when patients sought a second week’s sick leave due to a cold. Working with mild mental illness was considered to be doable over video consultation, since the patients were open and requesting help, assessment of mental status could be obtained, and no further action was needed.

… what I think is fun and works out well is dealing with skin conditions… then you’ve made a difference… your inner need to cure is satisfied… It could be Lyme disease rashes… if you get a good, clear picture of it… then it’s easy. (P 8)

… you usually base your assessment on the anamnesis in an area where if you’d had a dermatoscope you could have seen very quickly what it’s about. (P 7)

### The downside of being accessible online in relation to priorities in healthcare

Recommendations from the health centres to patients to seek the virtual clinic were problematic when the patients needed physical examinations which could not be carried out with video consultation. Some of the physicians sympathised with understaffed health centres, though it seemed that these health centres more often recommended patients to seek the virtual clinic because of its higher accessibility, including patients who were less suited to video consultation. When patients could not be fully helped through video consultation, one course of action was to use the visit time for what was possible. For example, a patient with severe menopausal symptoms who needed a physical examination in order to get medical treatment was instead given information and support.

In general, patients are often significantly better at triaging themselves than staff at understaffed units are. (P 9)

…I think it’s reasonable that the time the patient has booked with me should provide as much care to the patient as possible, even though the technique limits what I can ultimately do about the case. (P 7)

Doubts were expressed about the patient and social benefits of the virtual primary care clinic regarding priorities in healthcare on three main levels: the allocation of financial resources, the distribution of physicians, and the proper utilisation of skilled professionals’ time. Some physicians thought the virtual clinic was not always the right care unit; the consultation was not always medically motivated or could have been provided by other care professionals. The low position of the patients, at the virtual clinic, in the prioritisation of healthcare was pointed out. One of the participants felt that the increased accessibility of the video consultation implied a lower threshold for seeking help from a doctor. Health anxiety was problematic; for example, one story was told about a patient who had made repeated appointments and seemed to be reading on the internet while asking questions.

…care seekers get an additional loophole in healthcare. Some people make an appointment just because it’s accessible… Maybe advice from 1177 (healthcare advice service via telephone and internet) would have been enough … but here you can get directly to the doctor. (P 4)

From a socio-economic perspective, of course it’s unreasonable to put so much resources into what’s sometimes only qualified advice…it takes money from some other part of the healthcare system. After all, it’s patients who are very far down in the order of priority, often with very banal ailments. (P 5)

Some of the physicians considered that personal gains in terms of work environment and flexibility outweighed the potential disadvantages from a societal perspective. They also noted that the allocation of resources in healthcare was down to politicians and not physicians. Others considered the work meaningful because of the advantages for the patients who used the service.

… for my own challenge and development of my medical skills and also for the benefit of society, I might have preferred to work in a context that takes care of a sicker part of the population, but the advantages of online work in terms of work environment, working hours, and the opportunity to work from home outweigh it… (P 7)

## Discussion

Our results reveal diverse experiences of working with video consultation at a public virtual primary care clinic. Video consultation changes the basis for assessment of primary care patients, where parts of the consultation process as well as clinical experience and patient involvement are crucial. The physicians in this study had adapted their work procedure to video as a consultation medium and had adopted new functions beyond the traditional physician’s role. The flexibility of work time and space and the limited assignment improved their work situation and wellbeing. Video consultation was considered suitable for some tasks and some patients in primary care, though its easy availability was thought problematic in terms of medical prioritisation in healthcare.

### Strengths and weaknesses of the study

The qualitative method and a relatively broad research question proved to be appropriate, as the results revealed previously unknown aspects of the phenomenon. The choice of interview technique, avoiding leading and pre-formulated questions, also contributed to an increased reliability of the information being truly based on the participants’ own experiences. A major strength in this study is that the method supported descriptions close to the transcripts and gave rich narratives of participants everyday lives in general and the consultation process in particular. The heterogeneous group of participants was also a strength, contributing to information variety.

One weakness is that the method only gave us information from those who were perhaps more inclined to express positive attitudes because of their decision to participate in the study. Moreover, the study only looked at the physicians’ experiences of one type of virtual primary care practice, which is a limitation. However, the intention was to investigate one specific context: the situation in which the virtual clinic is part of the patient’s regular public primary care organisation. Since the physicians at the virtual clinic also worked at the health centres, they were able to compare these two ways of delivering primary care and hence contribute to a comprehensive perspective of primary healthcare.

The first author’s internal perspective from working at one of Närhälsan’s health centres might be a weakness, but led to a cautious approach and was also compensated by the second author’s external perspective.

### Findings in relation to other studies

Beginning and ending the consultation with a click on the computer, instead of in physical waiting rooms, was perceived to facilitate clinical focus and efficiency. Under optimal conditions, physicians equated the contact through the screen with the contact during an in-person visit, using terms such as ‘eye contact’ and ‘face to face’. These findings were unexpected, given that according to the literature an existing doctor-patient relationship is important both for the video consultation to be successful [14,23] and for patients to be willing to use the service [10,24]. However, visual cues have previously been described as benefits of video consultation, favourably building rapport and communication [14].

Patient-centred consultation, in particular eliciting the patient’s thoughts, concerns, and requests, was described as crucial for the video consultation. This corresponds to a model developed by Larsen and Risør, who also teach telephone consultation skills. The lack of an in-person meeting means that physicians may miss certain visual cues and are unable to conduct certain physical examinations. They therefore have to relate to the patient’s story, which is facilitated by hearing the patient’s own thoughts [25]; on this point, video consultation has similarities with telephone consultations. Our results indicate varied consultation methods depending on what the physicians perceived as effective communication. In a randomised crossoverstudy, primary care physicians reported no differences between in-person visits and video consultation in their ability to conduct histories [26]. Nevertheless, video consultations have been observed to be less patient-centred, with the physician dominating the conversation [27], giving fewer verbal responses, and understanding what was on the patient’s mind to a lesser extent [28].

The physicians considered video consultation suitable for patients with mild mental illness. There are studies from the 1990s implying that video as a communication medium makes patients with severe mental illness (schizophrenia) less verbally inhibited, which improved information exchange [29]. Nevertheless, our results indicate that the potential of video consultation for the treatment of mental illness could be limited by the severity of illness, as video consultation requires the patient to have a stable mental status in order to be able to interact in front of the screen.

The physicians had adapted to video as a consultation medium in a way that enabled some form of physical examination to take place, replacing parts of the traditional examination by a physician-guided patient self-examination. Although virtual examination requires the patient to be capable of assisting, these findings stand in contrast to the earlier viewpoint that video consultation might be unsuitable for patients in need of physical examination [11]. Physician-guided patient self-examination over video has been described in case reports earlier, such as for remote evaluation of a patient with appendicitis [30] and could be seen as a new type of patient involvement which allows a more advanced interaction between patient and physician. The randomised crossover study showed no substantial differences in clinical decision making between in-person visits and video consultations, but the physicians found the physical examination capabilities to be the least satisfying aspect of video consultation [12,26]. The coronavirus pandemic has accelerated the development of virtual care, and there are now guidelines for virtual examination of patients with Covid-19, at least [31].

Being the only professional involved with the patient during the video consultation meant that the physicians had to fulfil several functions (e.g. triage, helping with information technology) in addition to the traditional physician’s role. Some of these findings resemble earlier findings from specialist outpatient care, where video consultation brought new roles and tasks [23] as well as flexibility and convenience for the physicians [32]. What the present study adds is the concern of the physicians that their medical competence might not be fully used because of their multifunction. This study also elucidates the advantages of the flexibility and the regulated assignment at the virtual primary care clinic in reducing workload, decreasing stress, and giving more control over the work situation. This is, from what we know, not described before.

The results indicate that open access for booking a video consultation challenges the order of priority in healthcare. An increased use of healthcare was considered a consequence, as seen in the case of privately-provided video consultation [16,33]. One study showed comparable follow-up rates between video consultations and in-person visits of commercially insured patients, but the majority of video consultations were for self-healing conditions [15] and the need for follow-up should reasonably depend on severity of illness and disease burden. Users of privately-provided video consultation, not connected to health centres, have been found to have fewer primary care contacts in the past [8,16] and low disease burden [15,16], leaving the question of whether these video consultations represent new utilisation of healthcare. Furthermore, elderly patients with chronic diseases have expressed fears of reduced access to healthcare if younger patients overuse the system because of lack of triage with online booking [34]. In order to perform primary care prioritisation based on patients’ care needs, a well-functioning triage system has been pointed out as necessary for virtual care services as video consultation [35]. At present, the impact of virtual care services on the consumption of healthcare is not well studied [4].

Providing video consultation as an additional service to the health centres within the same organisation was considered to facilitate both the virtual care process and patient safety. There could be risks associated with virtual care as a parallel system, isolated from other healthcare, where patient responsibility is unclear and continuity and information transfer are difficult [35]. If two parallel care systems – virtual and traditional – continue to develop separately, the consequence might be increased workload at the health centres due to lowered staffing and more demanding tasks as a consequence of the digital recruitment of both doctors and easy cases. Providing traditional and virtual care within the same care organisation can prevent further development of two parallel care systems.

### Meaning of the study

Consultation skills and physician-guided patient self-examination have the potential to increase the information exchange over video, allowing physicians to treat patients virtually. Video consultation facilitates primary care for patients who have difficulty visiting the health centres, provided that they have complaints suitable for video consultation and are capable of assisting in the examination. Combining clinical work at a health centre with virtual care could be a way of reducing the workload of the individual physician and ensuring medical competence in virtual primary care. Providing video consultations within the same organisation as the patient’s regular health centre is considered to be favourable for patient care and safety, by the participants in the study.

The consultation skills taught today are based on traditional in-person visits. Consequently, there is a need to study how video as a consultation medium affects the process, content, and outcome of the consultation, as well as the importance of consultation skills for video consultation. The findings of such research should be implemented in physician education. The extent to which assessment of patients can be based on physician-guided self-examination should also be further studied.

It is not yet clear whether virtual care will improve the general work situation for primary care physicians in the long term. There is a need to study the patient and societal benefits of video consultation at virtual primary care clinics with open access, as well as virtual care in general. Adapting virtual care to meet the increased need of healthcare in the population based on medical priorities is a particular challenge at present.

## Conclusion

For the physicians, video consultation induced changes in the basis for assessment of primary care patients. The limitations on informational exchange demanded an extended form of patient involvement founded upon consultation skills, clinical experience and new skills for virtual examination. Combining virtual care with traditional general practice has the potential to reduce the workload for the individual physician and ensure medical competence in virtual primary care. Video consultation experienced suitable in some situations, but easy access to it expressed problematic in terms of medical prioritisation in healthcare.
